# Supplementary material for: Recycling of Uridylated mRNAs in Starfish Embryos
Source: Biomolecules. 2024 Dec 16;14(12):1610. doi: 10.3390/biom14121610 (PMC11674185; doi:10.3390/biom14121610)
Supplement: Supplementary file 1 [file biomolecules-14-01610-s001.zip › biomolecules-3331825-SM/biomolecules-3331825-supplementary.pdf]

Supplementary Materials

Table S1. Primers used for RT-PCR.

Figure	Target	Primer Name	Sequence (5′ to 3′)
Figure 1	starfish *cyclin B*	sfcycB_F	GAAAGCCCATCTGATCAAGC
		TGIRT_R	CAGACGTGTGCTCTTCCGAT
Figure 2	starfish *Rps29*	sfRps29_F1	TCACATCTGCCCCAAAGCAT
		PCR-R&RT	GTCTCTAGCCTGCAGGA
Figure 4	starfish *Rps29*	sfRps29_F2	TGTGTGGCAATCGTCATGGA
		TGIRT_R	CAGACGTGTGCTCTTCCGAT
Figure 4	exogenous mRNA	exo_tag_F	AAAGATCTGCAGCGTGCGTCA
		TGIRT_R	CAGACGTGTGCTCTTCCGAT
Figure 6	Starfish Rps 29 5′ UTR	40S 5′ RACE R	TCGGTTGGTGTTGCTGCGCT
Figure S2	*sfRpl27a*	sfRpl27a_F	AAGCCAAGTTCTTCAGCCGA
		PCR-R&RT	GTCTCTAGCCTGCAGGA

Table S2. Primers used for PCR to construct expression vectors and for qPCR.

Oligo DNA	Sequence (3′ to 5′)
psfcycB_vector_F	GTTCCATCCTACGAATTCCT
psfcycB_vector_R	TTTAACTTTTATTTACAAAGACTGACA
psfcycB_A25_insert_F	AAATAAAAGTTAAATGGAAATAAAAAAAAAAAAAAAAAAAAAAAAAC-GCATGTTCCATCCTACGAA
psfcycB_A25_insert_R	TTCGTAGGATGGAACATGC
psfRps29_WT_vector_F	TGGCGGCCGCTCTAGAAAGATCTGCAGCGTGCGTCAAACCTACTCTAGAAGCAGCTGG
psfRps29_WT_vector_R	GTTTTCAAGCAGATCTGGTCATT
psfRps29_WT_insert_tem-plate	GACCAGATCTGCTTGAAAACAAAAAAAAAAAAAAAAAAAAGAGACGCTAGAACTAGTG-GATCCCCC
psfRps29_WT_insert_F	GACCAGATCTGCTTGAAAAC
psfRps29_WT_insert_R	ATCCACTAGTTCTAGCGTCTC
psfSGK_vector_F	TGCAGGTCGACTCTAGAGGA
psfSGK_vector_R	AGAAACTTTCTTTTTATTAGGAGCAGATAC
psfSGK_long_insert_F	AAAAAGAAAGTTTCTTCACATTCTAAAAAAAAAAAAAAAAAAAAAAAAAGTCTTCTG-CAGGTCGACTCTA
psfSGK_long_insert_R	TAGAGTCGACCTGCAGAAGAC
psfSGK_short_insert_F	GTTTCTTCACATTCTAAAAAAAGTCTTC
psfSGK_short_insert_R	TAGAGTCGACCTGCAGAAGACTTTTTTT
psfSGK_nc1_insert_F	GTTTCTTCACATTCTAAAAATTTTTTTTAAAATTAAGTTTAATTTTTATTATAAAAAAAAA-GAAAAGAAAAAAAAATAAAAAAAAAAAAAAAAAAGAAAAAATTTAAAAAAAAAAAAAAAAA AAAAAAAAAAAAAAGTCTTCTGCAGGTCGACTCTA
psfSGK_nc1_insert_R	TAGAGTCGACCTGCAGAAGAC
psfSGK_nc2_insert_F	GTTTCTTCACATTCTAAAAAATTTTTTTTTTAAAATATTTTTTAA-TAAAAAAAAAAATTTTTATAATATTTTTTTAAAAAATTTAAAAAAAATAAATAAAAAATTTTTA AAATATATAATTTAAAAGTCTTCTGCAGGTCGACTCTA
psfSGK_nc2_insert_R	TAGAGTCGACCTGCAGAAGAC
18S F qPCR	GCGGCCGAAACGTTTACTTT
18S R qPCR	TTCCATGCTCTGCTGTCCAG
Cyclin B F qPCR	CTCAACAACGCACCCAAGT
Cyclin B R qPCR	TGAACTTGCTGCTGGCATAC
40S ribosomal protein S29 F qPCR	GGCAATCGTCATGGACTGAT
40S ribosomal protein S29 R qPCR	GTGAACTGCACCCTGAGAAA
sfRps29-GST_1F	CGCGGTGGCGGCCGCTCTAGGACCAAATTGTAAAATTTCTTTCTCT
sfRps29-GST_1R	TAACCTAGTATAGGGGACATATCCATCTTTTGAAATCCAATGTC
sfRps29-GST_2F	ATGTCCCCTATACTAGGTTATTGGA
sfRps29-GST_2R	AGCTGCTTCTAGAGTAGGTTTCATTTGGAGGATGGTCGCC
BsmBI-pBlue3'	ATCCACTAGTTCTAGCGTCTC
sfRps29-GST_3F	AACCTACTCTAGAAGCAGCTGG
sfRps29-GST_3R	CTAGAGCGGCCGCCACC
Nluc_FW2	CTCGTGGACGCCATCATGGTCTTCACACTCGAAG
Nluc_RV2	CTTCTAGAGTAGGTTTTACGCCAGAATGCGTTCG
vec_FW2	AACCTACTCTAGAAGCAGC
vec_RV2	GATGGCGTCCACGAGGTGC

Table S3. Statistical results of the targeted TAIL-Seq reads.

Sample Description	Target Gene	Endogenous/Exogenous	SRA Accession	# Raw Read Pairs	# Valid Read Pairs
Rps29 -1-MA	*Rps29* (40S)	Endogenous	DRR218044	159,138	113,040
Rps29 +1-MA 1.5 h	*Rps29* (40S)	Endogenous	DRR218045	212,874	172,224
Rps29 +1-MA 12 h Blastula	*Rps29* (40S)	Endogenous	DRR218046	124,150	75,876
Rpl27a −1-MA	*Rpl27a* (60S)	Endogenous	DRR218047	112,496	52,351
Rpl27a +1-MA 1.5 h	*Rpl27a* (60S)	Endogenous	DRR218048	117,322	64,800
Rpl27a +1-MA 12 h Blastula	*Rpl27a* (60S)	Endogenous	DRR218049	101,332	30,792
WT *Rps29* −1-MA 2 h Pro-I	*Rps29* (40S)	Exogenous (injected)	DRR218050	65,670	38,789
WT *Rps29* −1-MA 11 h Pro-I	*Rps29* (40S)	Exogenous (injected)	DRR218051	52,396	30,867
WT *Rps29* +1-MA 2 h PB	*Rps29* (40S)	Exogenous (injected)	DRR218052	76,771	58,632
WT *Rps29* +1-MA 5 h Morula	*Rps29* (40S)	Exogenous (injected)	DRR218053	49,119	33,505
WT *Rps29* +1-MA 11 h Blastula	*Rps29* (40S)	Exogenous (injected)	DRR218054	54,821	32,352
ΔPAS *Rps29* −1-MA 2 h Pro-I	*Rps29* (40S)	Exogenous (injected)	DRR218055	96,099	59,555
ΔPAS *Rps29* −1-MA 11 h Pro-I	*Rps29* (40S)	Exogenous (injected)	DRR218056	68,731	42,567
ΔPAS *Rps29* +1-MA 2 h PB	*Rps29* (40S)	Exogenous (injected)	DRR218057	77,342	52,094
ΔPAS *Rps29* +1-MA 5 h Morula	*Rps29* (40S)	Exogenous (injected)	DRR218058	88,182	64,476
ΔPAS *Rps29* +1-MA 11 h Blastula	*Rps29* (40S)	Exogenous (injected)	DRR218059	58,726	32,017
cyclin B TGIRT	*cyclin B*	Endogenous	DRR218060	171,337	148,143
cyclin B T4 RNA ligase	*cyclin B*	Endogenous	DRR218061	309,649	283,824

A

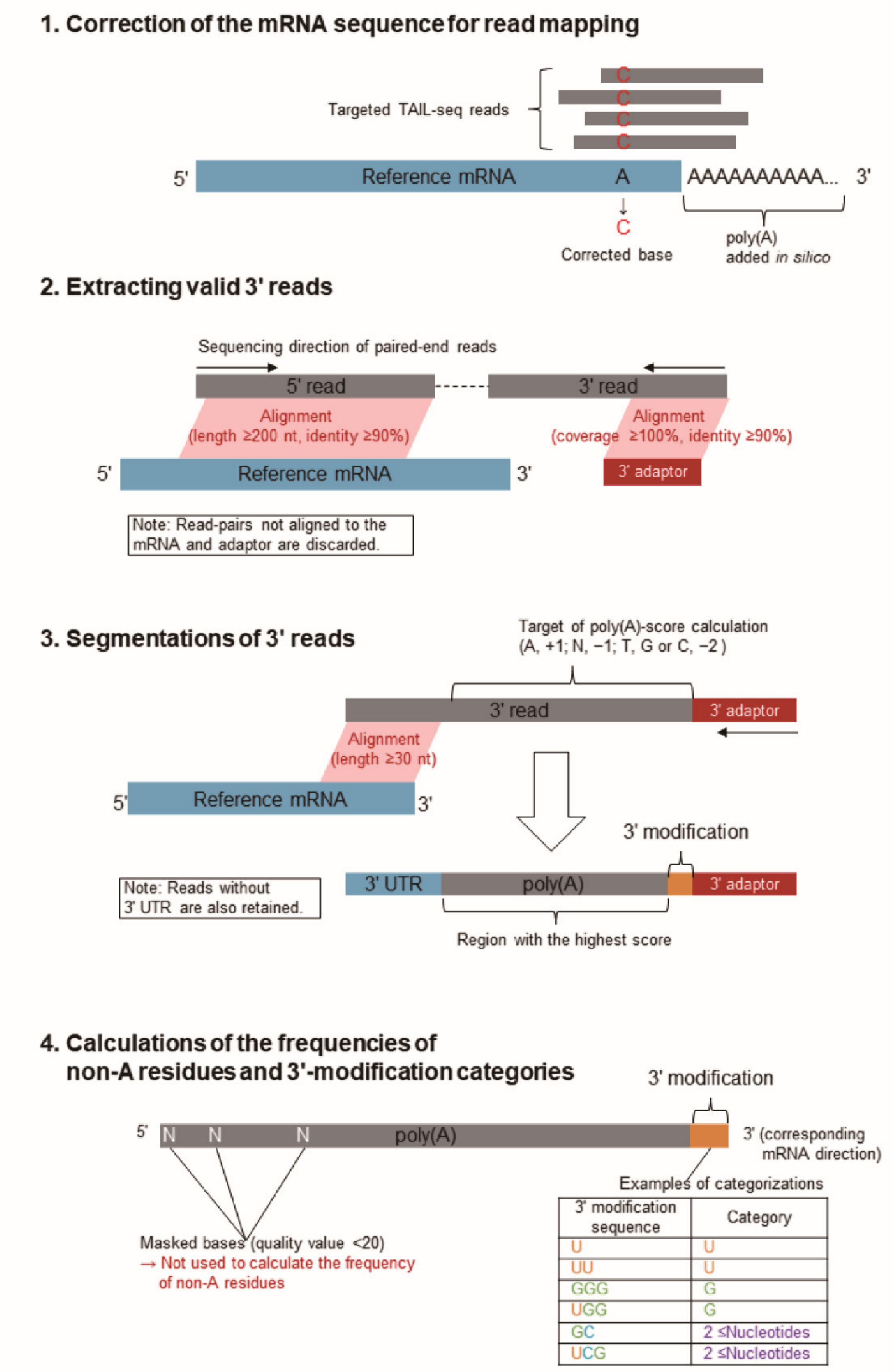
1. Correction of the mRNA sequence for read mapping
Targeted TAIL-seq reads
C
C
C
C
5'
Reference mRNA
A
AAAAAAAAAA...
3'
C
Corrected base
poly(A)
added *in silico*
2. Extracting valid 3' reads
Sequencing direction of paired-end reads
5' read
3' read
Alignment
(length ≥200 nt, identity ≥90%)
Alignment
(coverage ≥100%, identity ≥90%)
5'
Reference mRNA
3'
3' adaptor
Note: Read-pairs not aligned to the mRNA and adaptor are discarded.
3. Segmentations of 3' reads
Target of poly(A)-score calculation
(A, +1; N, −1; T, G or C, −2)
3' read
3' adaptor
Alignment
(length ≥30 nt)
5'
Reference mRNA
3'
3' modification
Note: Reads without 3' UTR are also retained.
3' UTR
poly(A)
3' adaptor
Region with the highest score
4. Calculations of the frequencies of non-A residues and 3'-modification categories
3' modification
5'
N
N
N
poly(A)
3' (corresponding mRNA direction)
Masked bases (quality value <20)
→ Not used to calculate the frequency of non-A residues
Examples of categorizations
3' modification sequence
Category
U
U
UU
U
GGG
G
UGG
G
GC
2 ≤Nucleotides
UCG
2 ≤Nucleotides

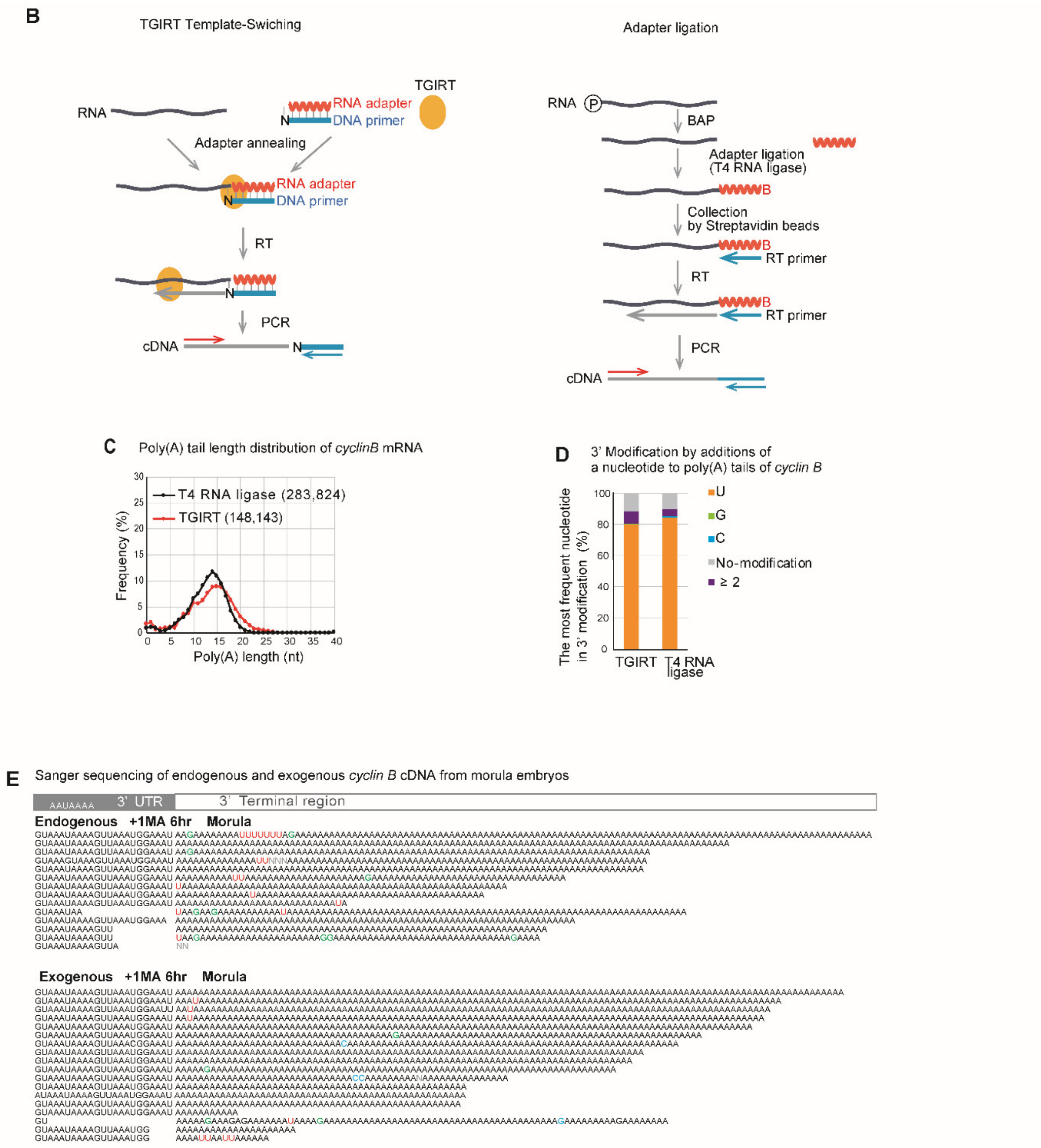

B
TGIRT Template-Swiching
Adapter ligation
RNA
RNA adapter
DNA primer
TGIRT
N
Adapter annealing
RT
PCR
cDNA
RNA
P
BAP
Adapter ligation
(T4 RNA ligase)
B
Collection
by Streptavidin beads
RT primer
C
Poly(A) tail length distribution of cyclinB mRNA
T4 RNA ligase (283,824)
TGIRT (148,143)
Frequency (%)
Poly(A) length (nt)
D
3' Modification by additions of
a nucleotide to poly(A) tails of cyclin B
The most frequent nucleotide
in 3' modification (%)
U
G
C
No-modification
≥ 2
TGIRT
T4 RNA
ligase
E
Sanger sequencing of endogenous and exogenous cyclin B cDNA from morula embryos
AAUAAAA
3' UTR
3' Terminal region
Endogenous +1MA 6hr Morula
Exogenous +1MA 6hr Morula

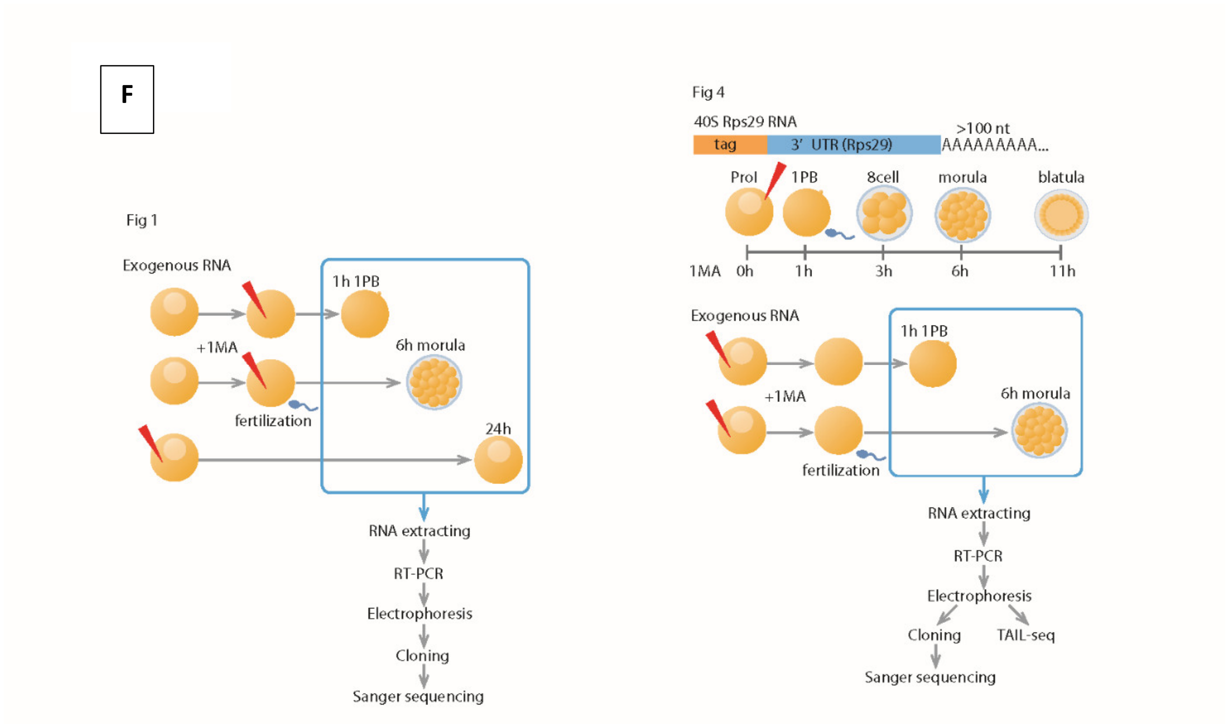

Figure S1. Experimental schemes, TAIL-Seq, and Sanger sequences. (**A**) Schematic model of the analysis of targeted TAIL-Seq. Details are provided in the Materials and Methods. (**B**) Experimental scheme of the TGIRT template-switching reaction (left panel) and adaptor ligation (right panel). Left panel: The DNA primer for TGIRT has a single nucleotide 3′ overhang with an equimolar mixture of A, T, G, and C, which can base pair with the 3′ end nucleotide of the target RNA. Reverse transcription with this enzyme, which is a TGIRT template-switching reaction, forms cDNA. Right panel: Biotinylated 3′ adaptor was ligated by T4 RNA ligase to the 3′ end of the mRNA, followed by reverse transcription to produce cDNA (Ochi, H. and Chiba, K. 2016 [1]). PCR was conducted using a gene-specific primer and 3′-adaptor primer. These two methods yielded identical results, as shown in B and C (below). The template-switching reaction by TGIRT showed good efficiency, requiring 30 oocytes ((**A**), left panel), whereas the method of ligating adapters using T4 RNA ligase ((**A**), right panel) showed poor reaction efficiency, requiring 100 or more oocytes. (**C**) Distribution of poly(A) tail lengths of *cyclin B* mRNA in oocytes without 1-MA treatment. The results obtained by the TGIRT template-switching reaction (red) were identical to those obtained by the T4 RNA ligase method (black), when targeted TAIL-Seq of *cyclin B* mRNA was conducted. Relative frequencies (Y axis, %) were calculated by dividing the number of detected reads carrying the indicated poly(A) lengths by the total number of reads containing poly(A) tails. The numbers of reads are shown in parentheses. (**D**) Relative frequencies of the most frequent nucleotide in additional modifications at the 3′ end of *cyclin B* mRNA poly(A) tails. Using each *cyclin B* mRNA read, the most frequent nucleotides, U, G, and C, were determined. Relative frequencies (Y axis, %) were calculated by dividing the number of reads with the most frequent nucleotides by the total number of reads. No modification; neither poly(A) tails nor additional modifications were present at the end of poly(A) tails. ≥2; two or more nucleotides comprised the most frequent nucleotides in the mRNA. (**E**) Sequencing results of the 3′ terminal region of endogenous and exogenous *cyclin B* mRNA from morula (6 h after 1-MA treatment). (**F**) Workflow of Figures 1 and 4.

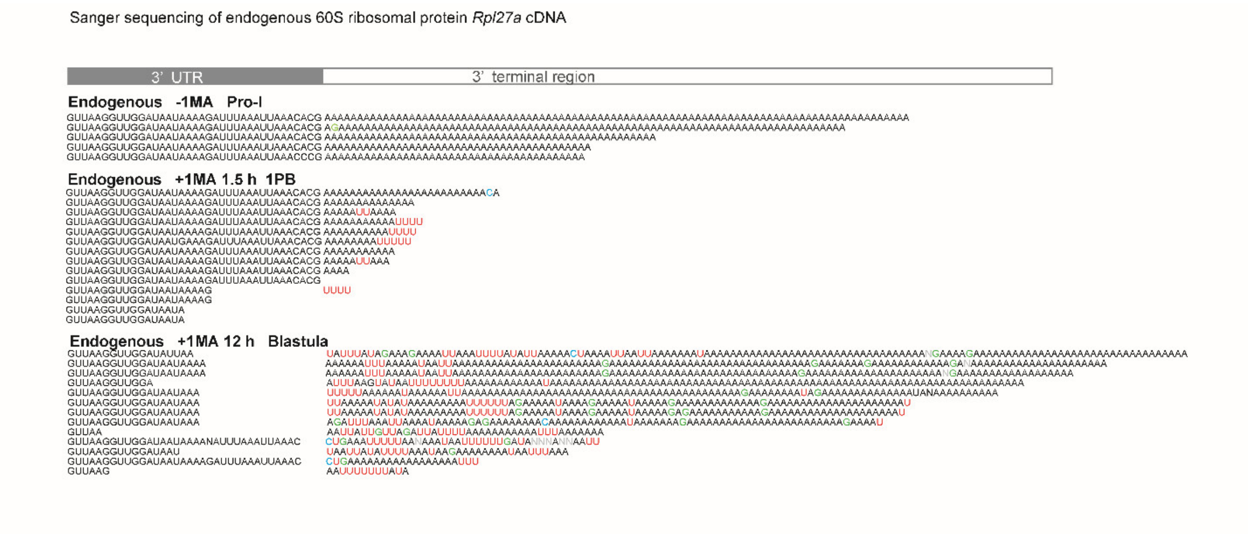

Figure S2. Sequencing results of the 3′ terminal region of the endogenous maternal *Rps27a* mRNA from oocytes at Pro-I without 1-MA treatment [Endogenous −1-MA], stimulated oocytes following first polar body formation 1.5 h after 1-MA treatment [Endogenous +1-MA 1.5 h], and blastula 12 h after 1-MA treatment [Endogenous +1-MA 12 h].

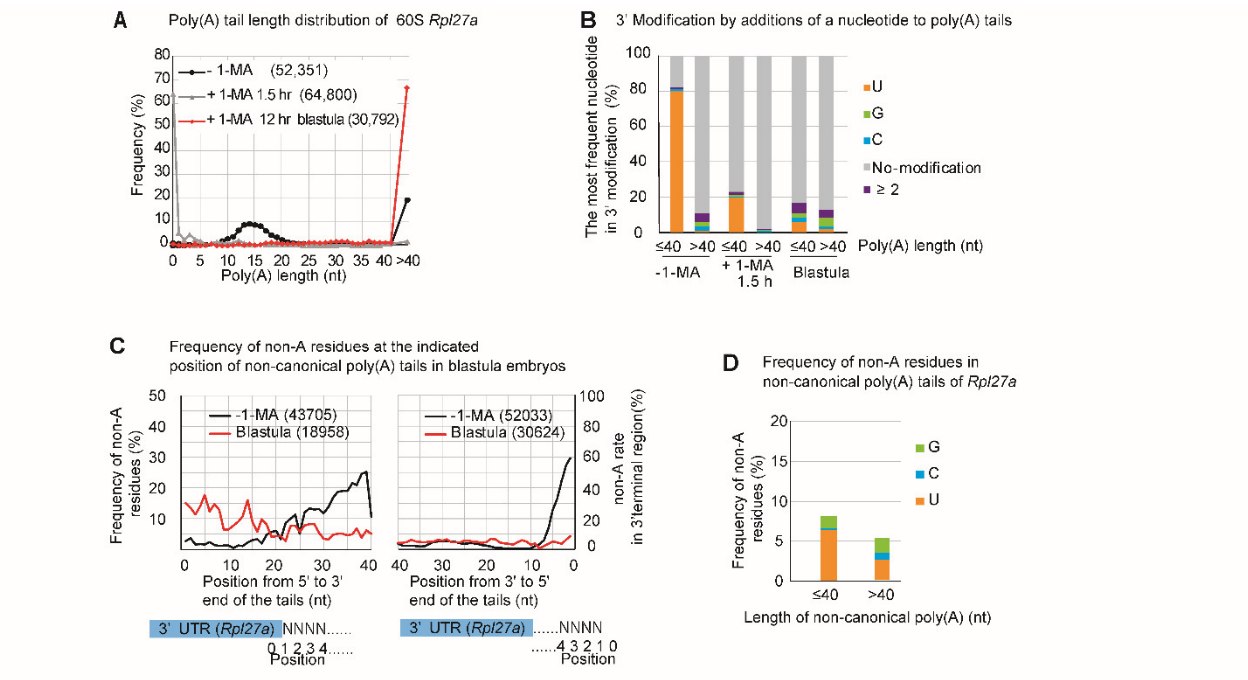

Figure S3. Targeted TAIL-Seq of *Rpl27a* mRNA. (**A**) Distribution of the poly(A) tail lengths of *Rpl27a* mRNA from oocytes with (+1-MA) or without 1-MA treatment (−1-MA), and from blastula embryos at 12 h following 1-MA treatment. Relative frequencies (Y axis, %) were calculated by dividing the number of detected reads carrying the indicated poly(A) lengths by the total number of reads containing poly(A) tails. Frequencies of poly(A) tail lengths of over 40 nucleotides are plotted on the right side (>40). The numbers of reads are shown in parentheses. (**B**) Relative frequencies of the most frequent nucleotide in additional modifications at the 3′ end of *Rpl27a* mRNA. Using each *Rpl27a* mRNA read, the most frequent nucleotides, such as U, G, and C, were determined. Relative frequencies (Y axis, %) were calculated by dividing the number of reads with the most frequent nucleotides by the total number of reads carrying the indicated lengths of poly(A) tails. The mRNAs carrying tail lengths of less than or equal to 40 nucleotides and those carrying over 40 were compared in each stage of oocytes and embryos. No modification; neither poly(A) tail nor additional modifications were present at the end of poly(A) tails. ≥2; two or more nucleotides comprised the most frequent nucleotides in the mRNA. (**C**) Distribution of relative frequencies of non-A residues in the tails of *Rpl27a* mRNA from blastula embryos and unstimulated oocytes. At the indicated position of the tails, the relative frequencies of the non-A residues (Y axis, %) were calculated by dividing the

number of reads carrying the non-A residues by the total number of reads. The distribution of frequencies of non-A residues is shown at the indicated position in tails from 5′ to 3′ (left panel) and from 3′ to 5′ (right panel). The numbers of reads are shown in parentheses. (**D**) Relative frequencies of non-A residues in the non-canonical poly(A) tails of *Rpl27a* mRNA. The relative frequencies of the non-A residues (Y axis, %) were calculated by dividing the number of non-A residues (G, C, and U) in the tails of all reads by the number of tail lengths of all reads that have indicated lengths of poly(A) tails. The mRNAs carrying tail length of less than or equal to 40 nucleotides, and over 40 were compared at the blastula stage of embryos.

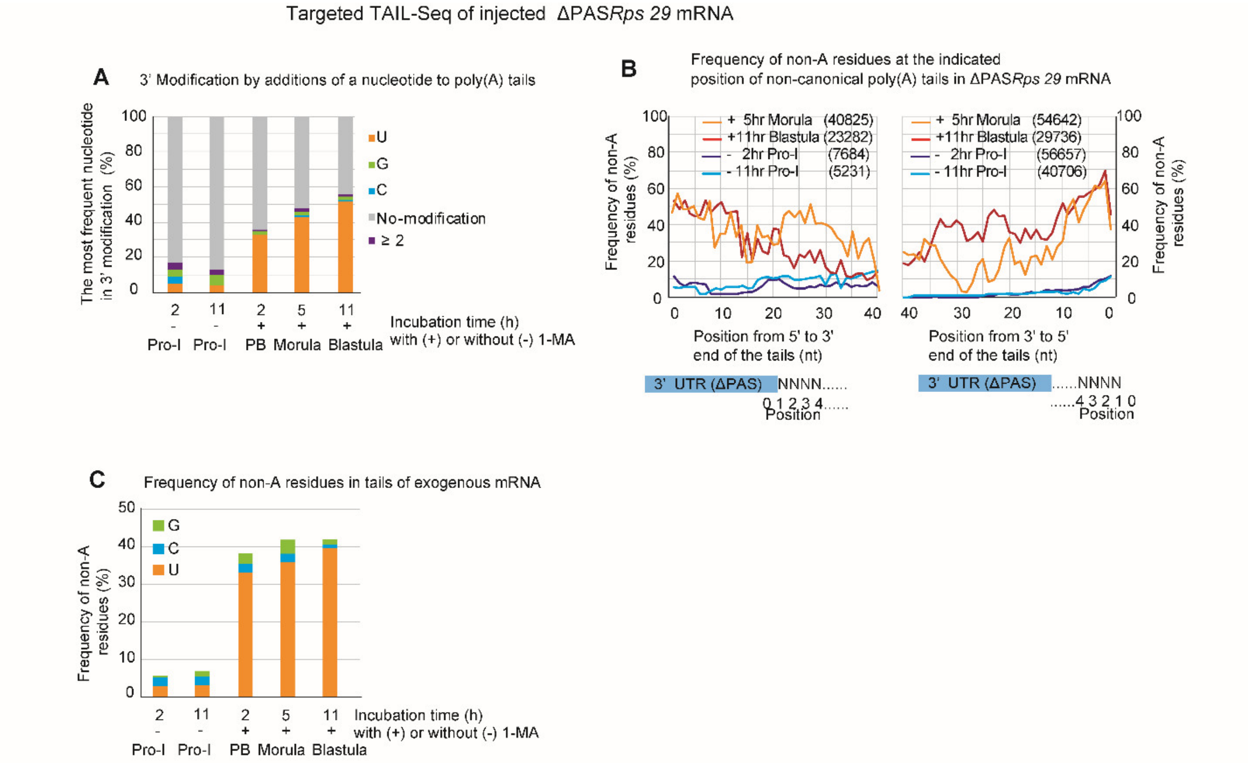

Figure S4. Targeted TAIL-Seq of Delta PAS *Rps29* mRNA. (**A**) Relative frequencies of the most frequent nucleotide in the additional modifications at the 3′ end of the exogenous Δ PAS *Rps29* mRNA. Using each exogenous Δ PAS *Rps29* mRNA read, the most frequent nucleotides, such as U, G, and C, were determined. Relative frequencies (Y axis, %) were calculated by dividing the number of reads carrying the most frequent nucleotide by the total number of reads from oocytes and embryos at the indicated time following 1-MA stimulation. No modification, either tail or additional modification was present in the mRNA. ≥2; over two nucleotides were the most frequent nucleotides in the mRNA. (**B**) Distribution of relative frequencies of non-A residues in the tails of exogenous Δ PAS *Rps29* mRNA from oocytes and embryos. At the indicated position of the tails, the relative frequencies of the non-A residues (Y axis, %) were calculated by dividing the number of reads carrying non-A residues by the total number of reads. The distribution of frequencies of non-A residues is shown at the indicated position in tails from 5′ to 3′ (left panel) and from 3′ to 5′ (right panel). The numbers of reads are shown in parentheses. Yellow, morula (5 h after 1-MA treatment). Red, blastula (11 h after 1-MA treatment). Purple, Pro-I oocytes without 1-MA stimulation (2 h after injection). Blue, Pro-I oocytes without 1-MA stimulation (11 h after injection). (**C**) Relative frequencies of non-A residues in canonical and non-canonical poly(A) tails of exogenous Δ PAS *Rps29* mRNA. The relative frequencies of the non-A residues (Y axis, %) were calculated by dividing the number of each non-A residue (G, C, and U) in the tails of all reads by the number of tail lengths of all reads.

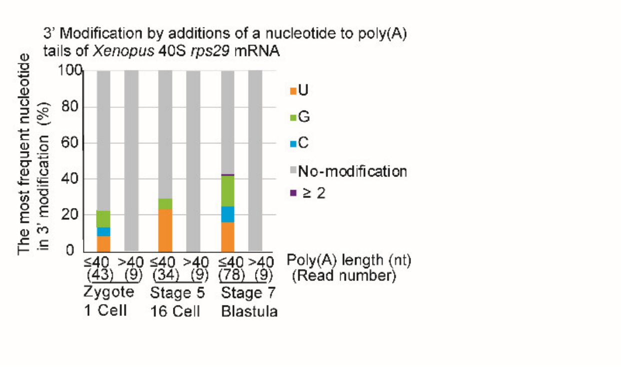

Figure S5. Relative frequencies of the most frequent nucleotide in additional modifications at the 3′ end of *Xenopus Rps29* mRNA. Using TAIL-Seq of *Xenopus* embryos (Chang et al. 2018), the most frequent nucleotides, such as U, G, and C, were determined. Relative frequencies (Y axis, %) were calculated by dividing the number of reads that carry the most frequent nucleotide by the total number of reads from oocytes and embryos at the indicated stage. No modifications, neither poly(A) tail nor additional modifications were present at the end of poly(A) tails. ≥2; two or more nucleotides comprised the most frequent nucleotides in the mRNA.

References

1. Ochi, H.; Chiba, K. Hormonal Stimulation of Starfish Oocytes Induces Partial Degradation of the 3′ Termini of Cyclin B mRNAs with Oligo(U) Tails, Followed by Poly(A) Elongation. *RNA* **2016**, *22*, 822–829. https://doi.org/10.1261/rna.054882.115.
