# Supplementary figures and images for: Recycling of Uridylated mRNAs in Starfish Embryos
Source: Biomolecules. 2024 Dec 16;14(12):1610. doi: 10.3390/biom14121610 (PMC11674185; doi:10.3390/biom14121610)

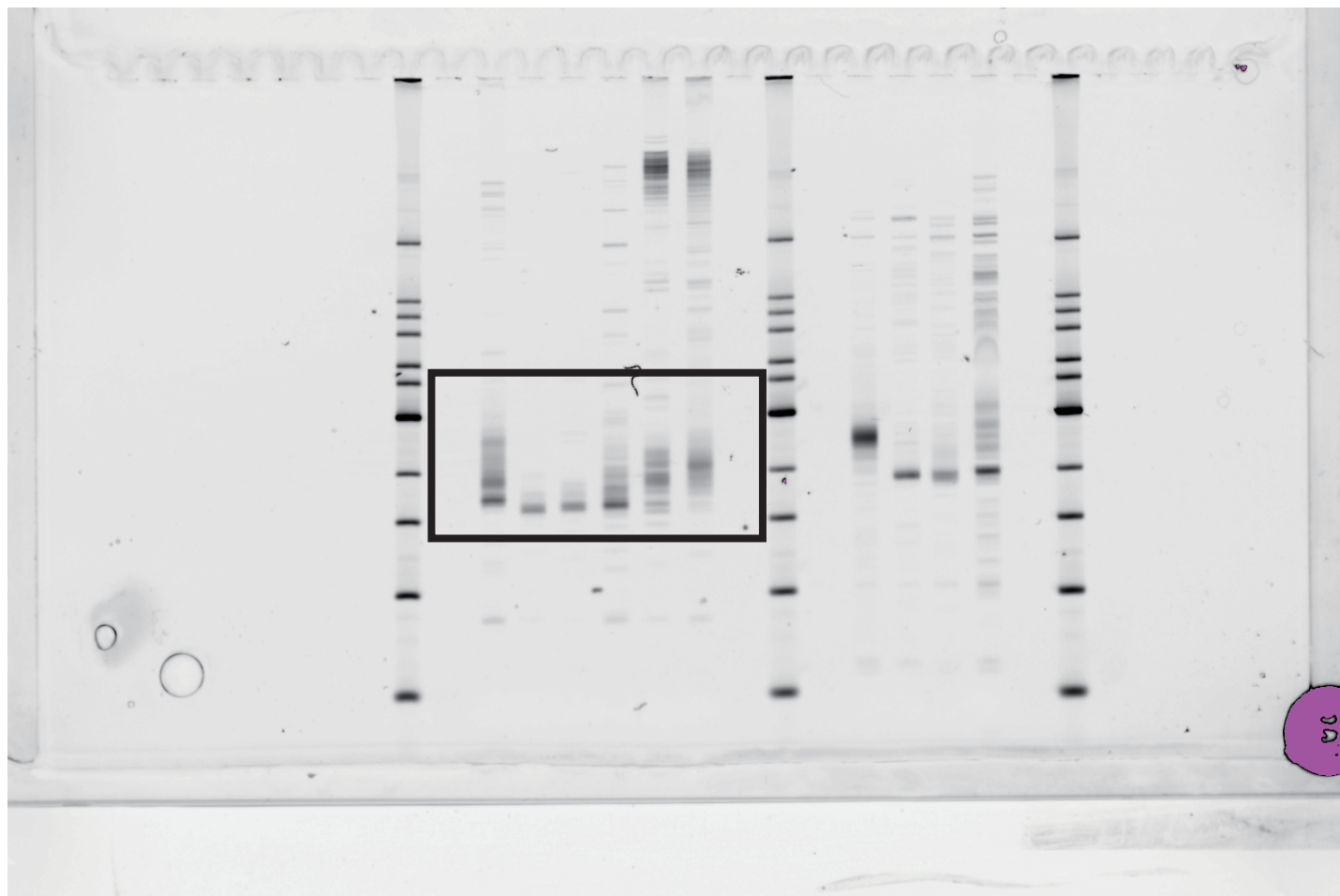

Supplement: Supplementary file 1 [file biomolecules-14-01610-s001.zip › biomolecules-3331825-SM/biomolecules-3331825-origna images/Fig. 2B original image.pdf]

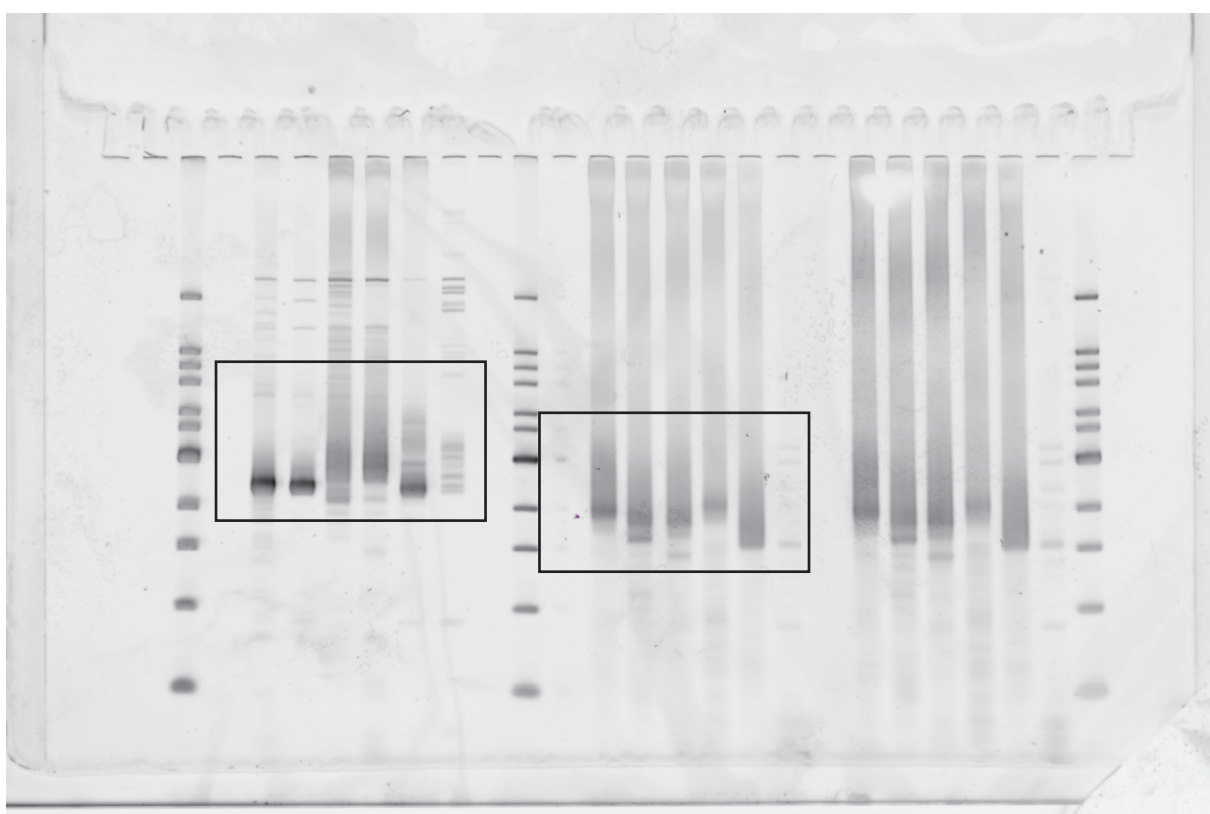

Supplement: Supplementary file 1 [file biomolecules-14-01610-s001.zip › biomolecules-3331825-SM/biomolecules-3331825-origna images/FIg.1 C original image.pdf]

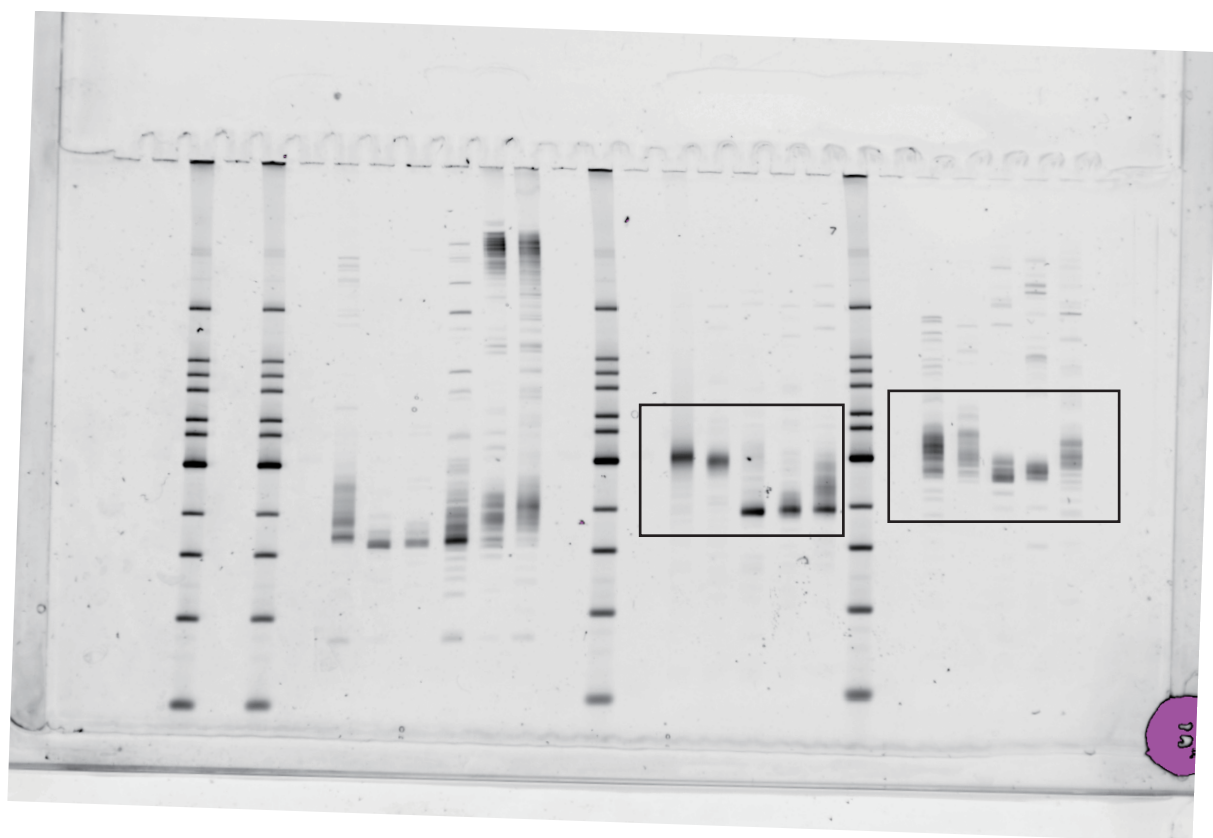

Supplement: Supplementary file 1 [file biomolecules-14-01610-s001.zip › biomolecules-3331825-SM/biomolecules-3331825-origna images/FIg.4b original image.pdf]

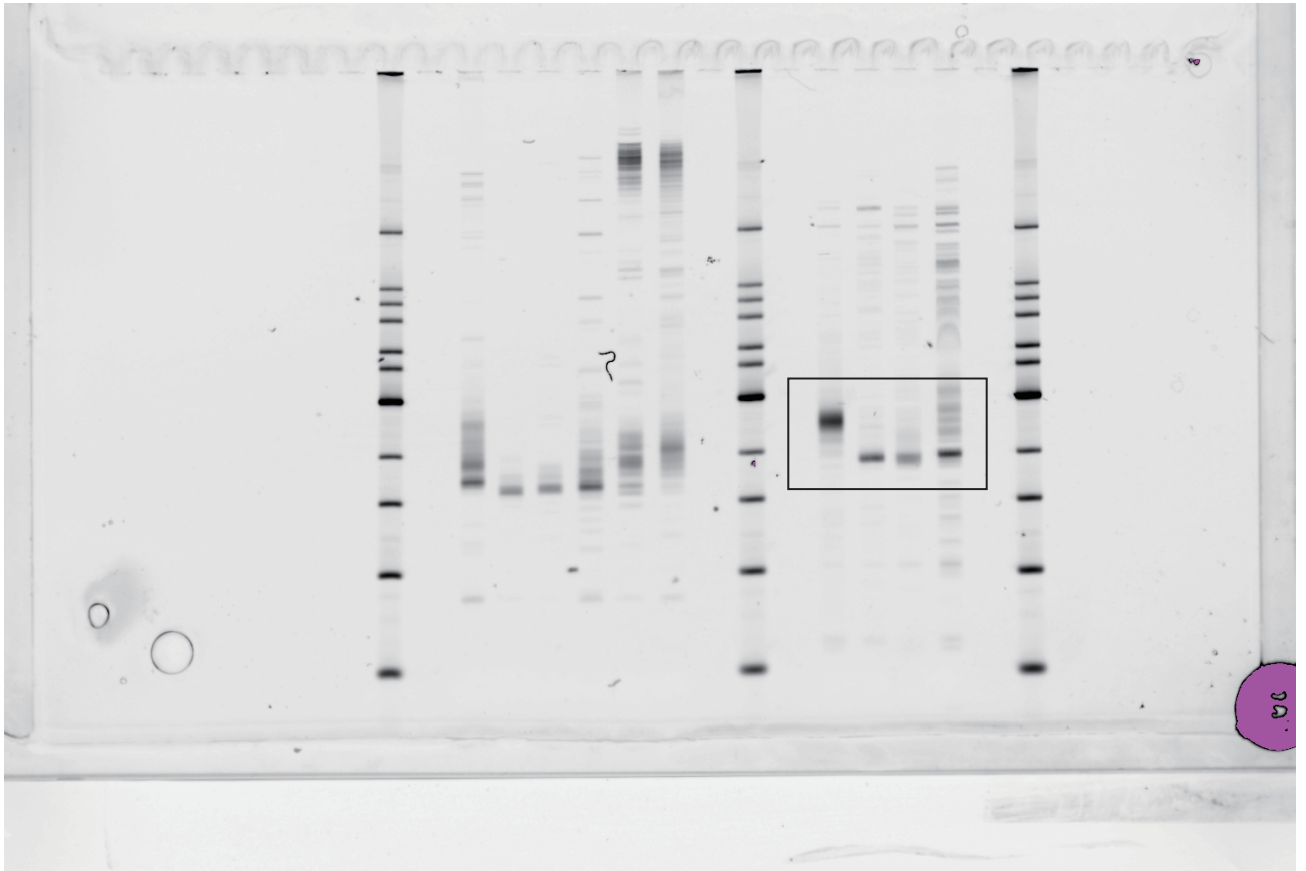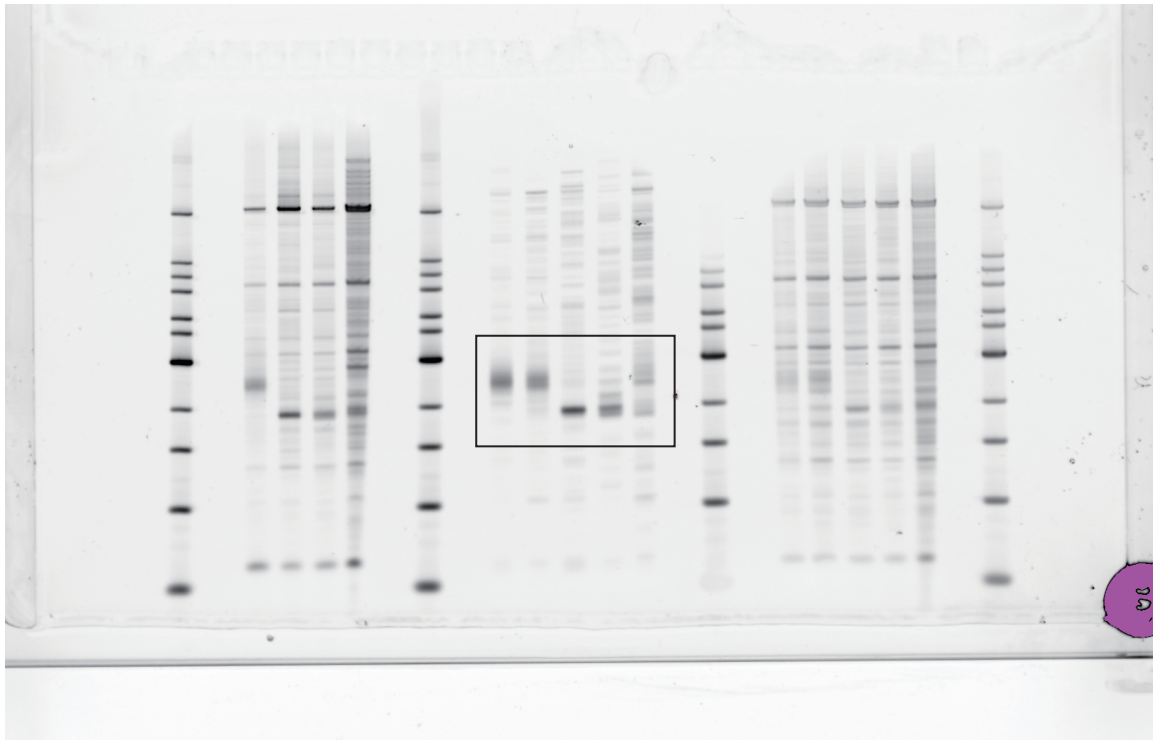

Supplement: Supplementary file 1 [file biomolecules-14-01610-s001.zip › biomolecules-3331825-SM/biomolecules-3331825-origna images/Fig.5b original image.pdf]

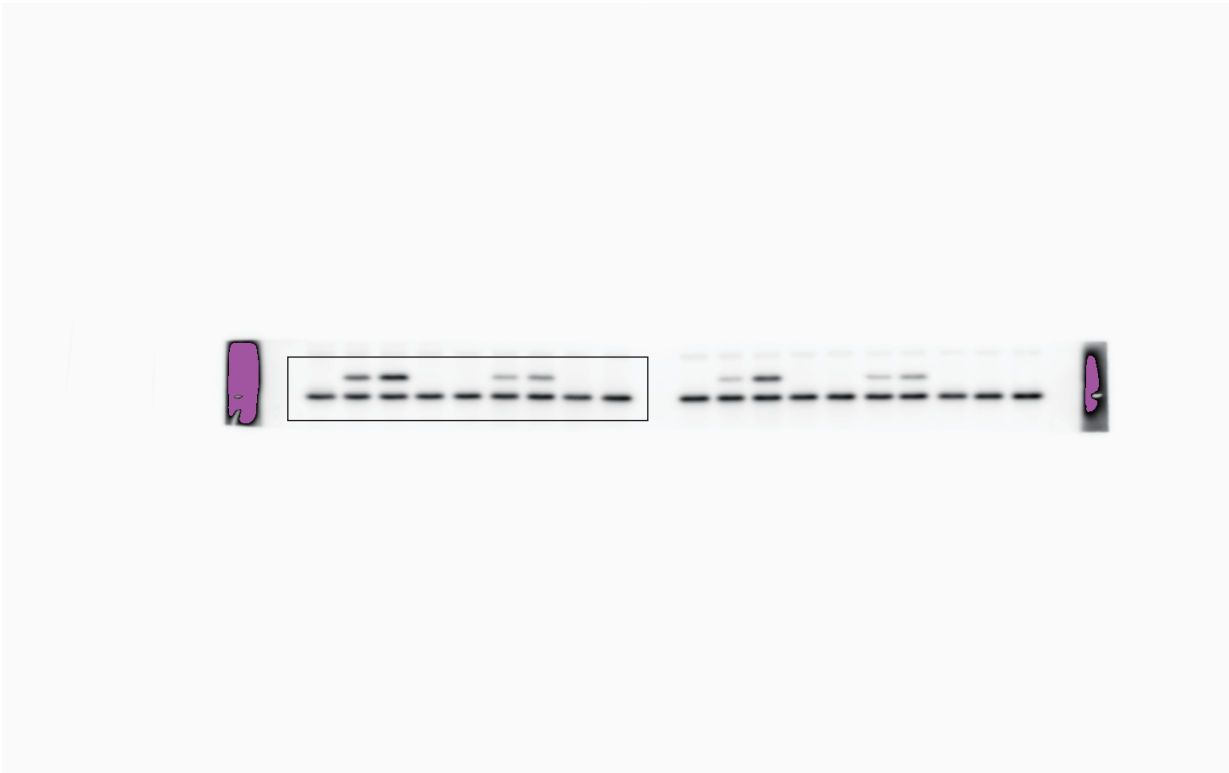

I

Supplement: Supplementary file 1 [file biomolecules-14-01610-s001.zip › biomolecules-3331825-SM/biomolecules-3331825-origna images/Fig.6 b original image1.pdf]

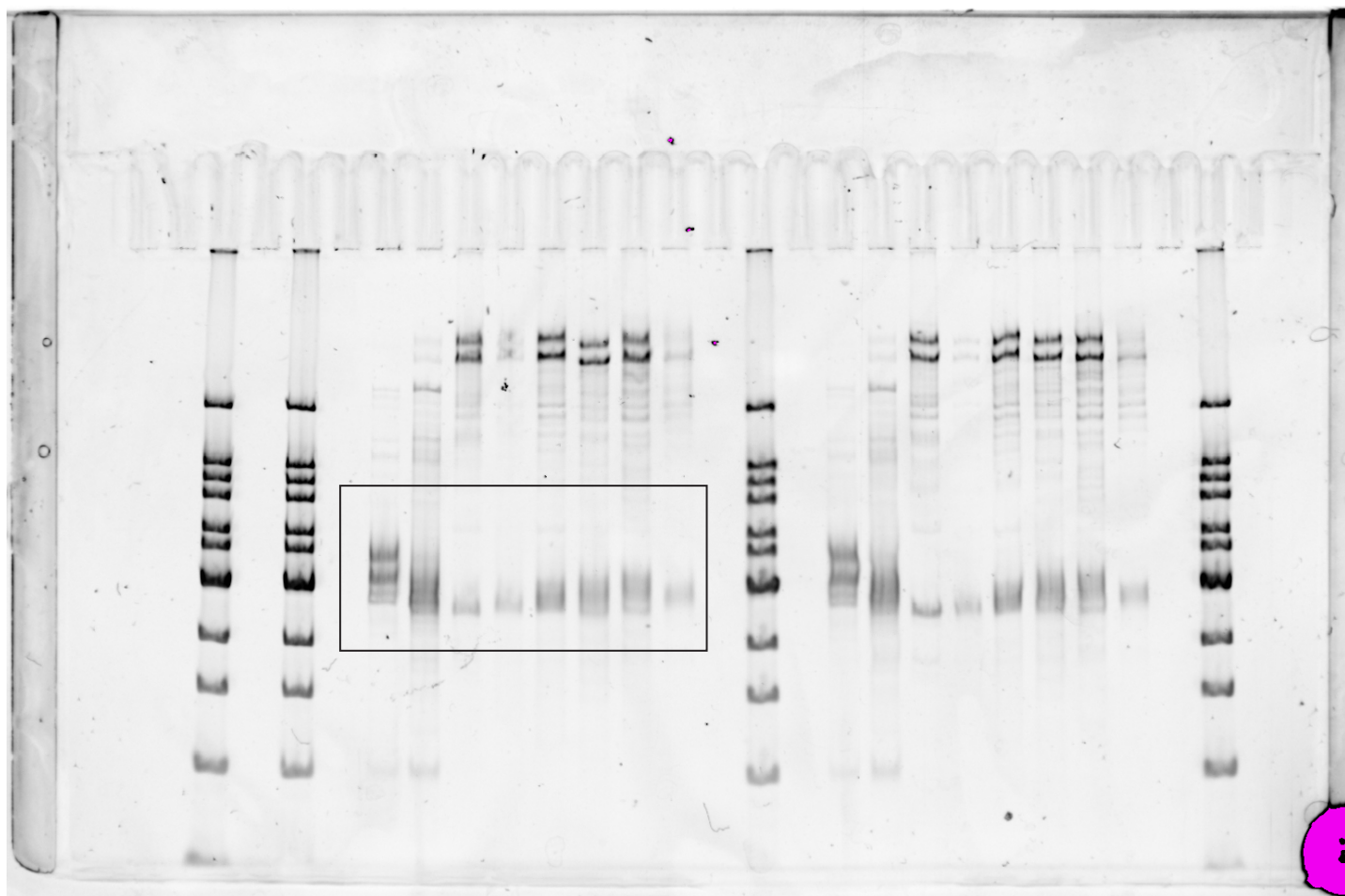

Supplement: Supplementary file 1 [file biomolecules-14-01610-s001.zip › biomolecules-3331825-SM/biomolecules-3331825-origna images/Fig.6E original image.pdf]
